# Narcolepsy and the Risk of Pregnancy Complications: Based on a Nationwide Healthcare System Database in South Korea

**DOI:** 10.1192/j.eurpsy.2025.2334

**Published:** 2025-08-26

**Authors:** T. W. Kim, S.-C. Hong

**Affiliations:** 1 Psychiatry, St. Vincent’s Hospital, The Cathoilc University of Korea, Suwon, Korea, Republic Of

## Abstract

**Introduction:**

Narcolepsy is known as an autoimmune disease which altered metabolic functions. It is believed that narcolepsy makes more pregnancy complications. However clinical evidence in narcolepsy patients, especially in pregnant women, is limited.

**Objectives:**

We aim to find out whether there is relationship between narcolepsy and pregnancy complications.

**Methods:**

We examined data from the South Korean nationwide health insurance claims database from 2010 to 2019. Out study included women narcolepsy patients who gave birth, and age- and sex- matched controls without narcolepsy. We estimated the odds ratio of narcolepsy with pregnancy complications and control group with pregnancy complications using multivariate logistic regression analysis.

**Results:**

Our study included 1,836 women with narcolepsy who gave birth and 28,796 women who gave birth without narcolepsy. We found that women with narcolepsy have a slightly high risk of preterm birth (OR, 1.191; 95% CI, 1.034-1.372). Patients with narcolepsy were at a significantly lower risk of spontaneous abortion, caesarean and gestational diabetes (OR, 0.763; 0.682-0.854, OR, 0.679; 95% CI, 0.560-0.824 and OR, 0.656; 95% CI, 0.556-0.774, respectively).

**Conclusions:**

This study is the first study about pregnancy complications in narcolepsy patients in South Korea. We found that preterm birth happened more in the patient with narcolepsy during pregnancy. But patient had lower risk of spontaneous abortion, caesarean, gestational diabetes compared to heath control group. These findings suggest that narcolepsy is not a definite risk factor for pregnancy complications. Further research is needed to investigate the reasons why narcolepsy patients had lower risk of spontaneous abortion, caesarean, gestational diabetes compared to health control.

**Disclosure of Interest:**

None Declared

